# Comparative Assessment of Bracket Bond Failure Rates of a Novel Chitosan-Based Orthodontic Primer: An In Vivo Split-Mouth Study

**DOI:** 10.7759/cureus.43177

**Published:** 2023-08-08

**Authors:** Deepika Katyal, Ravindra Kumar Jain

**Affiliations:** 1 Orthodontics and Dentofacial Orthopedics, Saveetha Dental College and Hospitals, Saveetha Institute of Medical and Technical Sciences, Saveetha University, Chennai, IND

**Keywords:** bonding, chitosan, bracket bond failure, :novel primer, orthodontics

## Abstract

Introduction

An unavoidable side effect of orthodontic fixed appliance therapy is the demineralization of the enamel surface surrounding the bracket at the adhesive and tooth interface due to a microgap formation, which serves as a nidus for biofilm growth. Due to this, it is advantageous to include antibacterial agents in orthodontic primers without affecting their clinical properties. The aim of this study was to compare the in vivo bracket bond failure rates of a novel chitosan-based primer with a conventional orthodontic primer.

Materials and methods

Fifty-four subjects and 1,080 brackets were included in this study. At the end of six months, 45 subjects and 960 brackets bonded using novel chitosan-based primers and conventional primers (Anabond, Anabond Stedman Pharma Research Pvt Ltd, Chennai, India). Each was evaluated for a bracketed bond failure rate. Descriptive statistics and Chi-square tests were used for statistical analysis.

Results

The results revealed that the bracket bond failure rate in Group 1 (novel chitosan-based primer) was 27 brackets (3%) and 23 brackets in Group 2 (conventional primer) (2.5%), with no statistically significant difference between the two groups (p>0.05). There was a statistically significant difference in the bracket failure rate between the maxillary arch (2%) and the mandibular arch (3.5%) (p<0.05).

Conclusion

Brackets bonded with the chitosan-modified novel orthodontic primer showed no statistically significant difference in bracket bond failure rate when compared to the conventional primer. Bond failure rates were higher in the mandibular teeth when compared to the maxillary teeth.

## Introduction

Orthodontic primers are made of unfilled resins that decrease the contact angle of the etched enamel, improving the adhesive's wetting and ability to penetrate the enamel's surface and ensuring a strong bond [1]. An unavoidable side effect of orthodontic fixed appliance therapy is the demineralization of the enamel surface surrounding the bracket at the adhesive and tooth interface [2]. The principal cause of this subsurface demineralization is the adherence of acid-producing cariogenic bacteria, primarily *Streptococcus mutans* [3]. Microgaps develop as a result of the adhesive contact disintegrating under repetitive loading, revealing the cured primer underneath, leading to the enamel and orthodontic adhesive interface acting as a nidus for biofilm growth and acid generation by the bacteria. Due to this, it is advantageous to include antibacterial agents in orthodontic primers [3]. In the past, numerous attempts to use different agents in orthodontic primers in an effort to reduce this demineralization have been made; for instance, fluoride and chlorhexidine have been added to orthodontic primers [4,5].

Chitosan has high physical and chemical qualities, good biocompatibility, and antibacterial capabilities. It is also biodegradable [6]. Recent years have seen an increase in the use of chitosan derivatives as antibacterial agents [6]. Chitin, a polysaccharide that is widely available, is used to make chitosan. The increased reactive capabilities of chitosan are caused by a number of functional groups that are present in the structure of the polymers and have cationic properties [7]. Past research has evaluated the mechanical properties of chitosan-containing varnishes and orthodontic adhesives as well as their antibacterial properties. According to the findings of these investigations, chitosan was added to orthodontic primers and composites to provide an antibacterial effect in the oral environments of patients receiving fixed appliance therapy [7, 8, 9]. In the past, a study evaluating the shear bond strength of a novel primer containing amorphous fluorinated calcium phosphate nanoparticles was carried out [10].

An ideal outcome of bracket bonding to any surface should result in an attachment that is strong enough to endure the forces of orthodontic treatment and mastication without dislodgement, while at the same time being safe enough to avoid damage to the surface during debonding following the end of the treatment [11]. An orthodontic primer acts as an interface between the enamel surface and the adhesive and therefore contributes significantly to the bond strength [11].

The aim of this study was to compare the in vivo bond bracket bond failure rate of novel chitosan-based primers with conventionally used orthodontic primers. The null hypothesis proposed was that the addition of chitosan as a beneficial antibacterial agent hampered the mechanical properties of the primer.

## Materials and methods

Study design and population

This prospective split-mouth clinical trial involved patients who visited the Department of Orthodontics and Dentofacial Orthopaedics at Saveetha Dental College and Hospital in Chennai over a period of 24 weeks. A priori power test was carried out with a power of 95. The sample size was calculated using the G*Power software, version 3.0.10 (Heinrich Heine University Düsseldorf, Dusseldorf, Germany), from a previously done study [11]. Institutional review board approval was obtained from the Saveetha Review Board (SRB) before starting the study (approval number was SRB/SDC/ORTHO-2005/23/035).

A total of 54 subjects between the ages of 15 and 38 were included in the study using a simple random sampling technique. The inclusion criteria included patients who had a complete complement of healthy, non-carious permanent teeth and required fixed appliance therapy in both arches without tooth extraction. The exclusion criteria included participants with craniofacial disorders, physical or mental disabilities, enamel hypoplasia, partially erupted teeth, or congenital enamel anomalies.

In this split-mouth investigation, two opposing quadrants of each individual were bonded using two types of primers. Group 1 was bonded using a novel chitosan-based primer, the details of which are mentioned in Table 1.

**Table 1:** The composition of the chitosan-modified primer Bis-GMA: bisphenol A-glycidyl methacrylate; PMGDM: pyromellitic glycerol dimethacrylate; HEMA: 2-hydroxyethyl methacrylate

Component	Percentage
Resin: Bis-GMA, PMGDM, HEMA	80%
Solvents: acetone and ethanol	15%
Additives: photoinitiators, inhibitors, stabilizers, etc.	+5%
Chitosan	0.1%–.25%

Group 2 was bonded using a conventional primer (Anabond, Anabond Stedman Pharma Research Pvt Ltd, Chennai, India). Written consent was obtained from all the subjects for their participation in the study.

**Figure 1:**
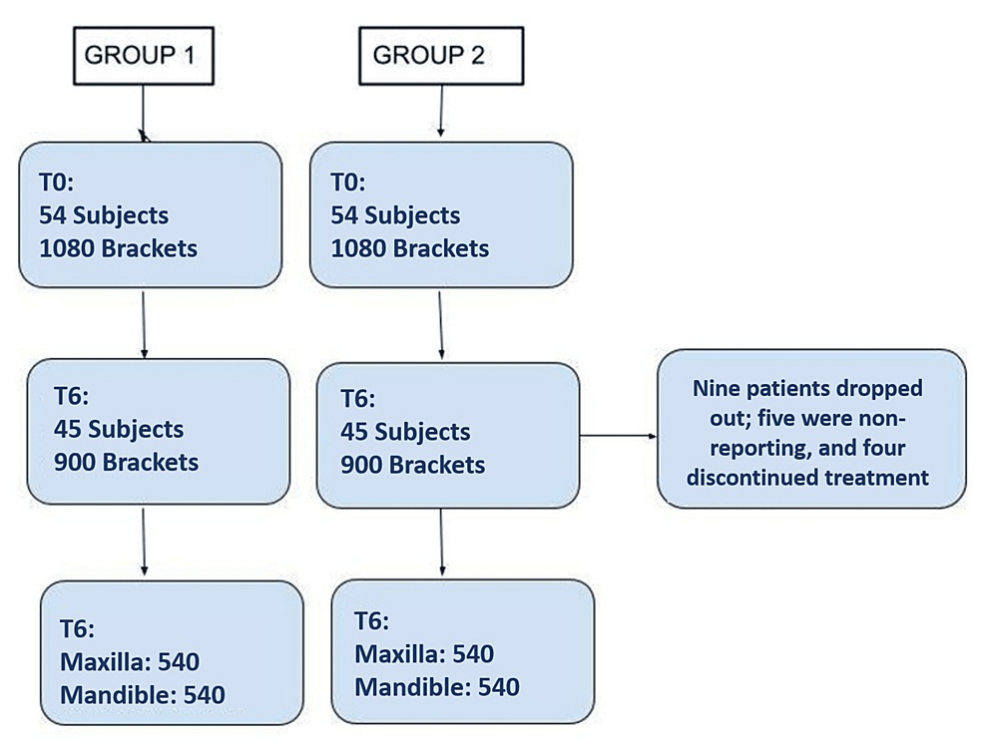
The consort flow diagram for Group 1 and Group 2 at the time of bonding (T0) and after six months (T6)

The consort flow diagram for Group 1 and Group 2 at the time of bonding (T0) and after six months (T6) is shown in Figure 1.

Bonding procedure

**Figure 2:**
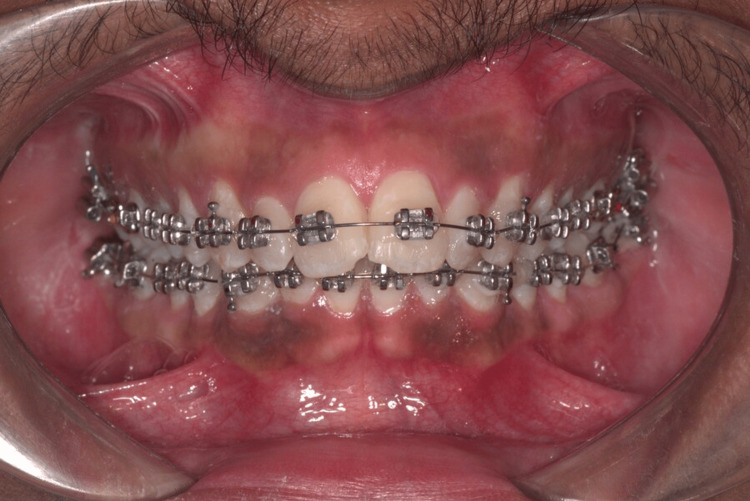
A figure depicting the bonding of the upper and lower arches

A complete oral prophylaxis was performed, and the teeth were polished with pumice. Cheek retractors, cotton wool rolls, and a low-volume suction evacuator with oil-free air were used to isolate the teeth. The labial surface of the teeth was etched for 15-20 seconds with 37% phosphoric acid (Anabond Eazetch, Anabond Stedman Pharma Research Pvt Ltd, Chennai, India), rinsed with water, and dried until the enamel appeared dull and frosty white. In all subjects, 0.022" maxillary transverse bioadaptation (MBT) metal brackets (3M Unitek Gemini, 3M India Limited, Bangalore, India) were bonded directly to the teeth on the center of the clinical crown using an MBT gauge. In subjects with an even registration number, the first and third quadrant teeth were bonded with chitosan-based primer (Group 1), and the second and fourth quadrant teeth were bonded with conventional primer (Group 2). In subjects with an odd registration number, the first and third quadrant teeth were bonded with conventional primer, and the second and fourth quadrants were bonded with chitosan-based primer. The primers were coated in a single layer on the labial surface and air dried for one to two seconds to aid in an even spread of the primer, then cured for five seconds. The brackets were bonded with the adhesive (Orthofix, Anabond Stedman Pharma Research Pvt Ltd, Chennai, India) and light-cured for three seconds (Woodpecker I-LED Plus curing light, Zhengzhou Linker Medical Equipment Co., Ltd., Zhengzhou, China) [12] (Figure 2).

A single operator, DK, bonded all teeth in a single appointment. Posterior bite turbos were used in cases of any occlusal interference. Subjects were given oral and written instructions about the maintenance of oral hygiene.

Observation and follow-up

For six months (T0: at the time of bonding, T6: six months post-follow-up), subjects were called back once every four weeks, and the operator, DK, noted bracket bond failures, if any, at each meeting. The following details were noted: 1. the tooth at which the debonding occurred; 2. the number of failed brackets; 3. the date of bond failure; 4. a possible explanation for the default. The rebonded bracket was excluded from the study once a bracket was debonded (Table 2).

**Table 2:** Sample characteristics T0: at the time of bonding; T6: post-six months; M: males: F: females

Time period	Number of subjects		Number of brackets assessed at T0, T6		Distribution of brackets by tooth type		
	F	M	F	M	Incisor	Canines	Premolars
T0	30	24	600	480	432	216	432
T6	25	20	500	400	360	180	360

Statistical analysis

Statistical analysis was performed using the Statistical Package for Social Sciences (SPSS) software version 13.0 (SPSS Inc., Chicago, USA). The Chi-square test with risk estimation was used to compare the likelihood of bracket bond failures between groups; the odds ratio was used to predict bond failures between the two groups; the arches involved (maxilla and mandible); the regions involved (anterior and premolars); and the sides involved (right and left). Kaplan-Meir analysis was done to evaluate the bond failure rate over six months.

## Results

A total of 45 patients and 900 brackets were evaluated after six months. A total of 50 brackets had debonded mid-treatment (5.5%). The details of the brackets that got debonded mid-treatment and the region of the bond failure have been shown in Table 3.

**Table 3:** Bracket failures in individual teeth T0: at the time of bonding, T1: first month, T2: second month, T3: third month, T4: fourth month, T5: fifth month, T6: sixth month Group 1: chitosan-based primer; Group 2: conventional primer

	Timeline	Incisors	Canines	Premolars
Group 1	T0	0	0	0
	T1	0	0	0
	T2	0	0	2
	T3	1	1	3
	T4	2	2	3
	T5	1	2	3
	T6	2	2	3
Group 2	T0	0	0	0
	T1	0	0	0
	T2	0	0	1
	T3	1	1	1
	T4	1	2	3
	T5	2	2	3
	T6	1	2	3

The results revealed that the bracket bond failure rate in Group 1 was 27 brackets (3%) and in Group 2 was 23 brackets (2.5%), with no statistically significant difference between the two groups (Table 4).

**Table 4:** Bracket bond failure (%) observed in both groups

Primers	Bonded (T0)	Evaluated (T1)	Failed	Failed%	Odds ratio	Chi-square	P-value
Group 1	540	450	27	3%	1.981	0.024	0.410
Group 2	540	450	23	2.5%			
Total	1080	900	50	5.5%			

There was a statistically significant difference in the bracket failure rate between the maxillary arch (2%) and the mandibular arch (3.5%) (p<0.05) (Table 5).

**Table 5:** Bracket failures (%) observed in the maxillary, mandibular, right, and left sides of the arches involve (posterior arch referring only to premolars)

Category	Characteristic	Bonded (T0)	Observed (T1)	Failed	Failed (%)	Odds ratio	Chi-square	P-value
Arch	Maxilla	540	450	18	2	1.64	0.031	0.001
	Mandible	540	450	32	3.5			
Teeth	Anterior	648	390	22	2.4	2.64	1.15	0.052
	Posterior	432	372	28	3.1			
Side posterior arch	Right	216	90	15	1.6	0.723	0.564	0.332
	Left	216	90	13	1.4			

**Figure 3:**
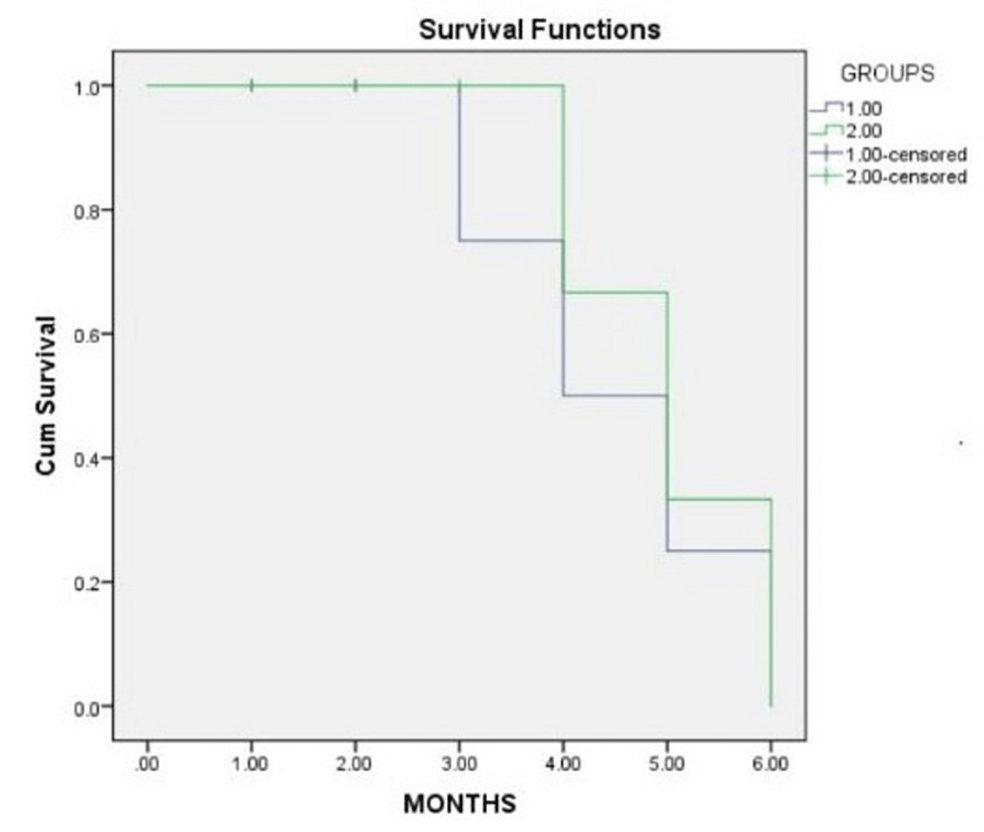
Survival rate graph of the two groups Group 1: chitosan-based primer; Group 2: conventional primer Cum: cumulative

Kaplan-Meir analysis was used to analyze the bond failure rate over a period of six months. There was no significant difference between the novel chitosan-based primer and the conventional primer during the six-month period of intervention. Even after six months of intervention, there was no significant difference between the two groups. Six months post-intervention, there was no difference in the survival rate between chitosan-modified primer and conventional primer (log-rank mantel cox p value =0.614) (Figure 3).

## Discussion

Enamel demineralization occurs as a result of the accumulation of cariogenic biofilm at the enamel adhesive interface [11]. Various attempts have been made to incorporate antimicrobial agents into orthodontic primers to combat demineralization and maintain the mechanical properties of the primer. The aim of this study was to evaluate the bracket bond failure rate of a novel chitosan-modified orthodontic primer. The results of this study revealed that there was no significant difference in the bracket bond failure rate between the novel chitosan-modified primer and the conventionally used primer, indicating that the addition of chitosan as an antibacterial agent did not affect the mechanical properties of the novel primer. Debonding of brackets on mastication of a hard food substance was reported as the most common cause by most patients included in this study. A statistically significant difference was found in the rate of debonding between the lower and upper arches, which could possibly be attributed to unwarranted forces exerted on the lower teeth during chewing. Molars were not considered in this study as banding of the molars was required in a few patients.

Chitosan is a potent antibacterial agent due to its property of binding to the cell wall and preventing DNA replication, leading to cell death [13]. It is also a strong chelating agent and binds to organisms, releasing toxins and inhibiting their growth [13, 14]. A study performed by Sukontapatipark et al. reported that microgaps of 10 to 15 μms form at the enamel orthodontic adhesive interface, which aids bacterial accumulation [15]. The acids secreted by these bacteria come into contact with the primer, and thus, the incorporation of antimicrobial agents like chitosan into the primer can help prevent bacterial activity and enamel demineralization.

A previous study was performed wherein the antibacterial, cytotoxic, and mechanical properties of the novel chitosan-based primer were assessed in vitro and showed promising results. The results of that in vitro study revealed that the novel primer showed good antibacterial properties against* Steptococcus mutans*, lesser cytotoxicity, and similar mechanical properties when compared with the conventional primer [15].

An in vivo study was performed to further substantiate that the addition of an antibacterial agent like chitosan did not hamper the mechanical properties of the primer. A study done by Tuma et al. reported that a newly developed calcium fluoride nanoparticle-containing orthodontic primer showed comparable mechanical properties to the control primer [16]. Another study done by Comert et al. also reported that a fluoride-releasing and rechargeable primer did not show a statistically significant difference when compared to the control primer with respect to clinical bracket failure rates [17]. To the best of our knowledge, no studies have been performed in the past with the incorporation of chitosan as an antibacterial agent in orthodontic primers, evaluating its in vitro and in vivo properties, and observing demineralization changes.

Limitations

The limitations of the study are the following: The follow-up period was limited to six months; however, fixed orthodontic appliance therapy usually takes a longer period for completion. The study was a single-center trial involving a single operator. Also, the growth pattern and force distribution levels based on occlusal contacts of the patients were not taken into consideration, which could possibly have an effect on the bracket bond failure rate. A long-term evaluation of the bracket bond failure rate in comparison to other commonly used primers can be done in the future.

## Conclusions

Brackets bonded with chitosan-modified novel orthodontic primer showed no statistically significant difference in bracket bond failure rate when compared to the conventional primer. Bond failure rates were higher in the mandibular teeth when compared to the maxillary teeth.
